# Synthesis and crystal structure of 5-{(*E*)-[(1*H*-indol-3-ylformamido)­imino]­meth­yl}-2-meth­oxy­phenyl propane-1-sulfonate

**DOI:** 10.1107/S2056989025002087

**Published:** 2025-03-11

**Authors:** Reham A. Mohamed-Ezzat, Benson M. Kariuki, Aisha A. K. Al-Ashmawy, Aladdin M. Srour

**Affiliations:** a Chemistry of Natural and Microbial Products Department National Research, Centre,Cairo Egypt; b School of Chemistry, Cardiff University, Main Building, Park Place, Cardiff CF10, 3AT, United Kingdom; c Department of Therapeutic Chemistry, National Research Centre, Dokki, Cairo, 12622, Egypt; Tokyo University of Science, Japan

**Keywords:** crystal structure, indole, sulfonate

## Abstract

In the title mol­ecule, C_20_H_21_N_3_O_5_S, the methyl­ideneformohydrazide and meth­oxy­benzene groups are almost coplanar, with the indolyl group being rotated farther from the plane. The mol­ecules in the crystal structure form chains parallel to the *a-*axis direction through N—H⋯O hydrogen-bonding inter­actions. Neighbouring chains are linked by N—H⋯O contacts to form a three-dimensional network.

## Abstract 2

The crystal structure is characterized by a three-dimensional hydrogen-bonding network. Chains formed through N—H⋯O contacts are linked by additional inter­molecular bonds to complete the structure.

## 1. Chemical context

**Figure 1:**
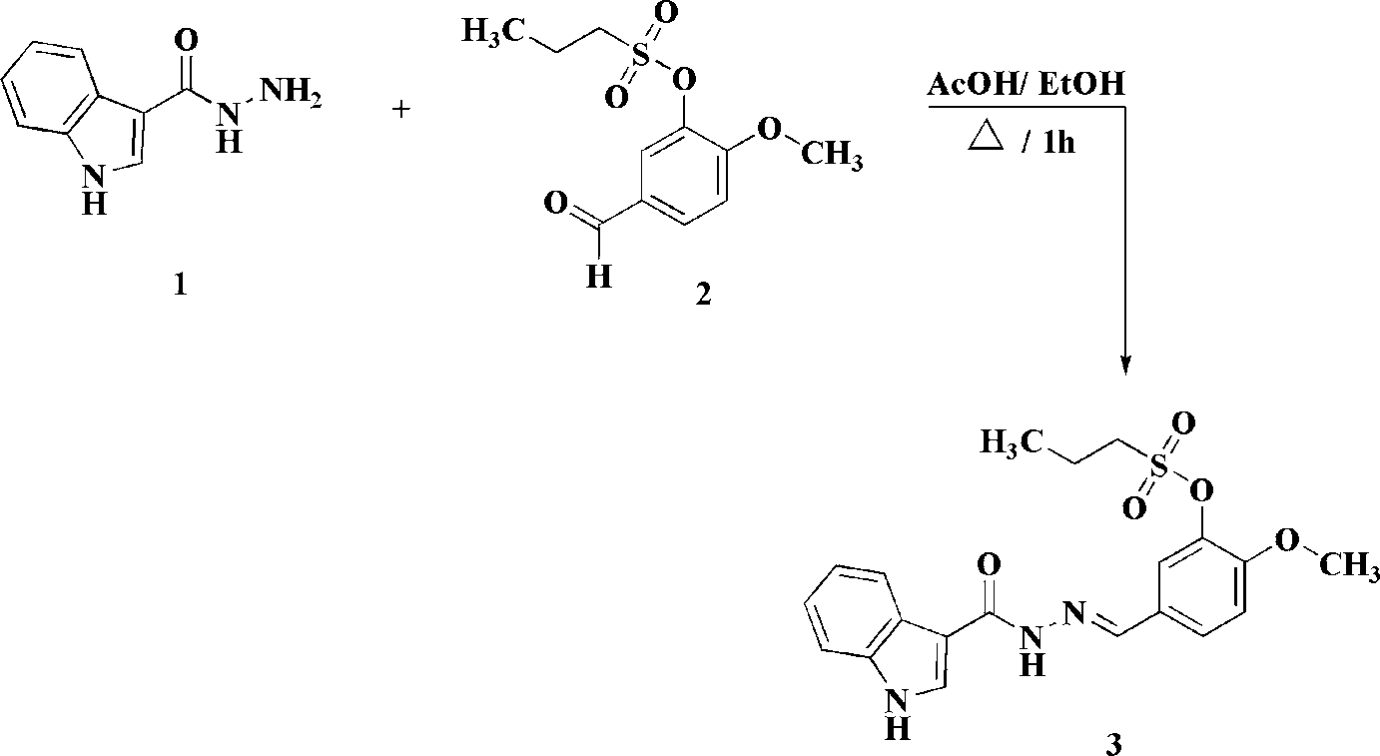
Synthesis of the novel title compound (**3**), which incorporates two bioactive moieties, indole and sulfonate, linked through a hydrazine linker.

Indole-based compounds are important structural motifs found in numerous natural products and serve as key scaffolds in many clinical drugs, including anti­cancer agents, anti­viral drugs, and non-steroidal anti-inflammatory agents (de Sa Alves *et al.*, 2009 ▸; Suzen, 2017 ▸). They also have various applications in biomedical research (Varun *et al.*, 2020 ▸; Facen *et al.*, 2024 ▸). As a result of their unique ability to mimic peptide structures and inter­act with enzymes, indole-based scaffolds are crucial in drug discovery (Kaushik *et al.*, 2013 ▸; Ubeid *et al.*, 2012 ▸; Citarella *et al.*, 2023 ▸). Recent advancements in drug discovery have driven the development of synthetic strategies to incorporate bioactive indole moieties into new mol­ecules. Similarly to indole-based compounds, sulfonate derivatives have recently shown a wide range of pharmacological effects, such as anti­microbial, anti­cancer, and anti­viral activities (Mohamed-Ezzat & Elgemeie, 2024*a* ▸,*b* ▸; Mohamed-Ezzat *et al.*, 2022 ▸, 2023*a* ▸, 2024*a* ▸,*b* ▸). Conjugates that containing both sulfon­ate and indole moieties have demonstrated significant potency as inhibitors of various biological targets, such as carbonic anhydrase, tubulins, phosphatidylinositol 5-phosphate 4-kinase (PI5P4K), MET tyrosine kinase (Pingaew *et al.*, 2021 ▸), butyrylcholinesterase (BChE) (Omar *et al.*, 2023 ▸), and HIV protease inhibitors (Batool *et al.*, 2024 ▸). In line with our research on developing synthetic approaches for bioactive heterocycles (Mohamed-Ezzat & Srour, 2024 ▸; Mohamed-Ezzat *et al.*, 2023*b* ▸,*c* ▸), we have designed and synthesized a novel compound featuring a hydrazone scaffold. Recognizing the broad potency of hydrazine-based derivatives (Elgemeie & Mohamed, 2014 ▸; Mohamed-Ezzat & Elgemeie, 2023 ▸; Mohamed-Ezzat *et al.*, 2023*c*,*d* ▸; Ragab *et al.*, 2024 ▸), the newly synthesized compound incorporates a conjugation of two bioactive moieties, indole and sulfonate, linked through a hydrazine linker (Fig. 1 ▸).
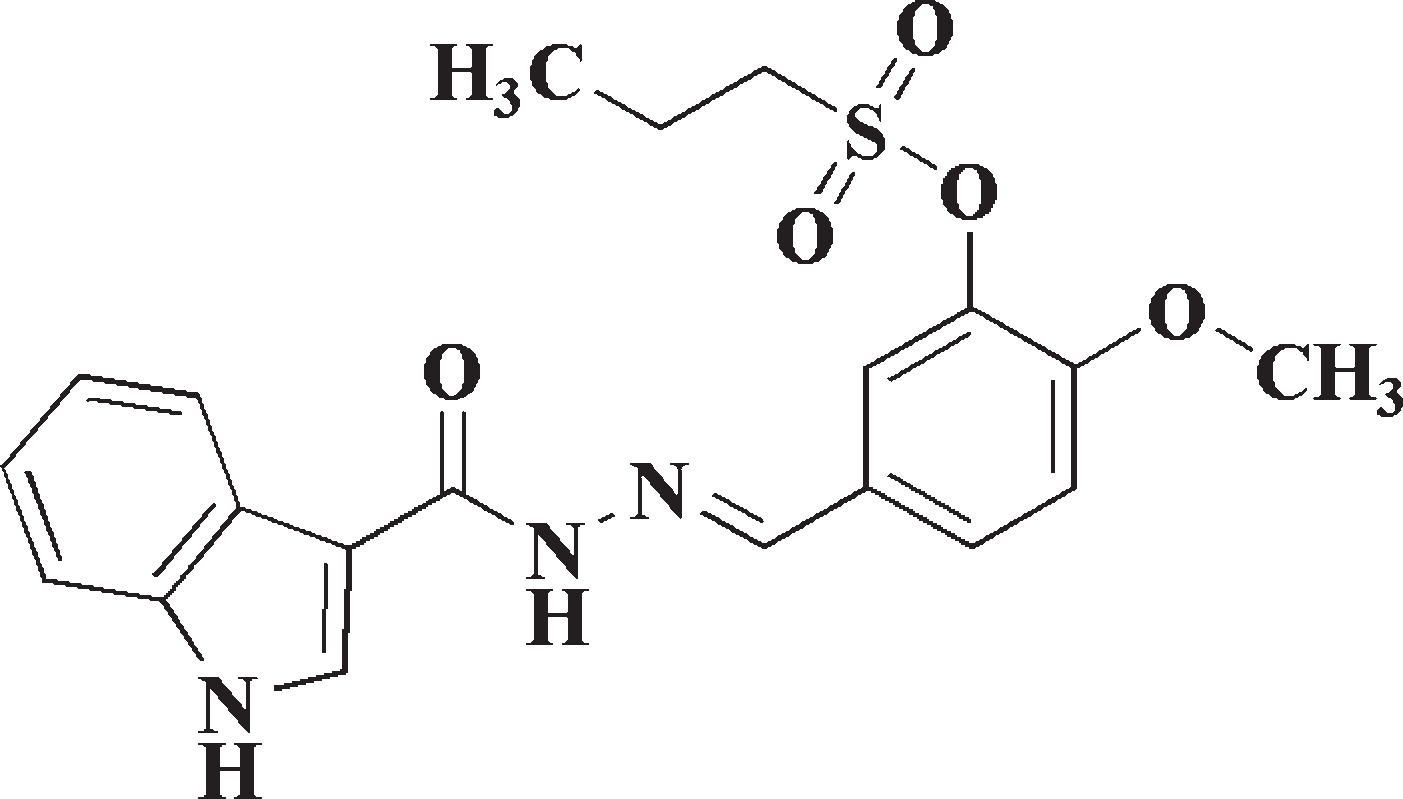


## 2. Structural commentary

**Figure 2:**
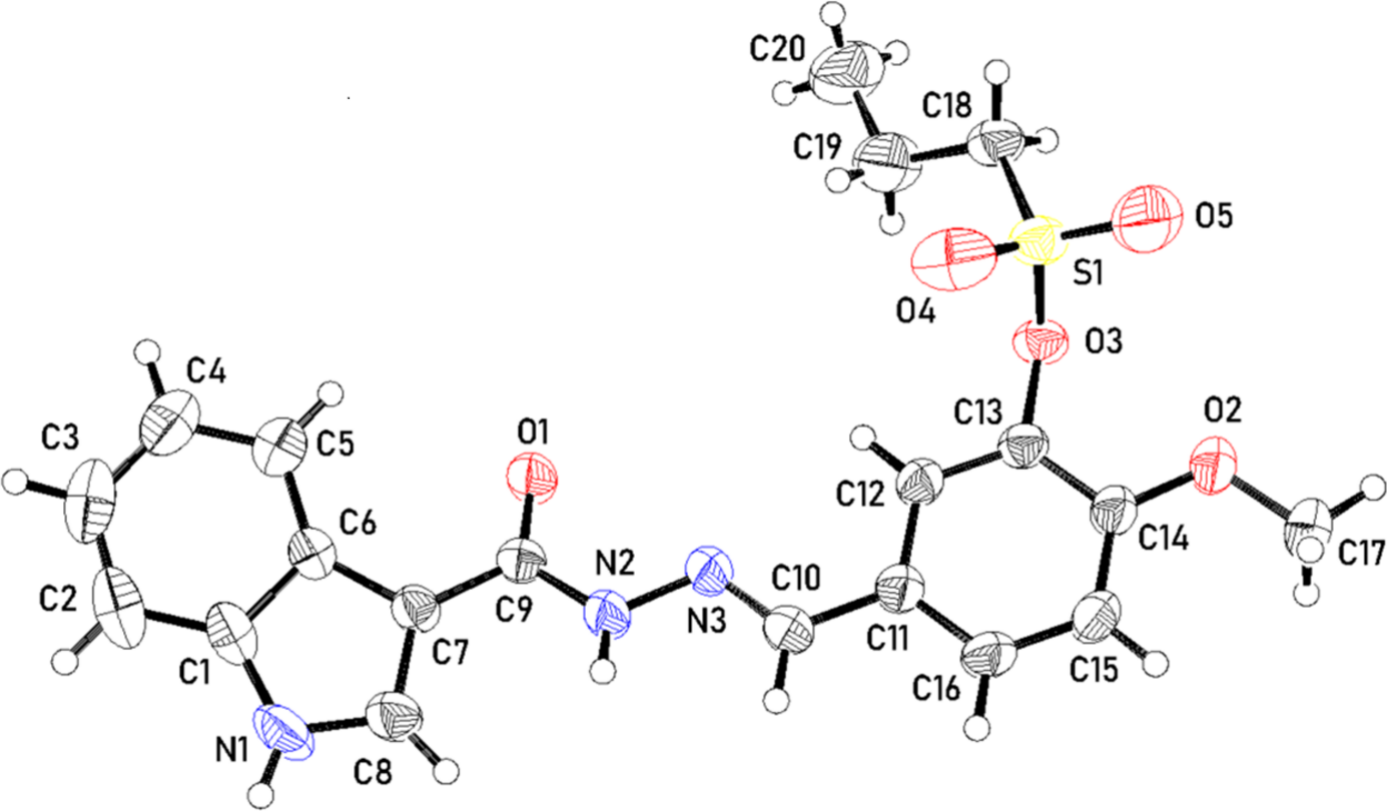
The mol­ecular structure of the title compound showing 50% probability displacement ellipsoids.

The asymmetric unit consists of one mol­ecule of 5-((*E*)-(1*H*-indole-3-carboyl­imino)­meth­yl)-2-meth­oxy­phenyl propane-1-sulfonate (Fig. 2 ▸). The mol­ecule comprises three planar fragments, namely indolyl (IND; C1–C8, N1), methyl­ideneformohydrazide (MFH, C9, C10, N2, N3, O1), and meth­oxy­benzene (MEB, C11–C17, O2) groups. In addition, the mol­ecule has a propane­sulfonate group (C18–C20, S1, O3–O5) with a nearly *trans* S1—C18—C19—C20 torsion angle [168.2 (4)°]. In the mol­ecule, the methyl­ideneformohydrazide and meth­oxy­benzene groups are almost coplanar with a MFH/MEB twist angle of 13.67 (17)°. A similar conformation is also observed in the structure of (*E*)-2-meth­oxy-*N′*-[4-meth­oxy-3-(4-methyl­phenyl­sulfon­yloxy) benzyl­idene] benzohydrazide ethanol solvate hemihydrate(Chen & Yu, 2006 ▸) where the twist angle is 7°. In the mol­ecule of the title compound, the indolyl group is rotated farther from the plane defined by MFH and MEB with a IND/MFH twist angle of 25.93 (14)°. The geometry is similar to that of *N′*-(2-hy­droxy­benzyl­idene)indole-3-formyl­hydrazine (Li *et al.*, 2024 ▸), which has a corresponding twist angle of 18.1°.

## 3. Supra­molecular features

**Figure 3:**
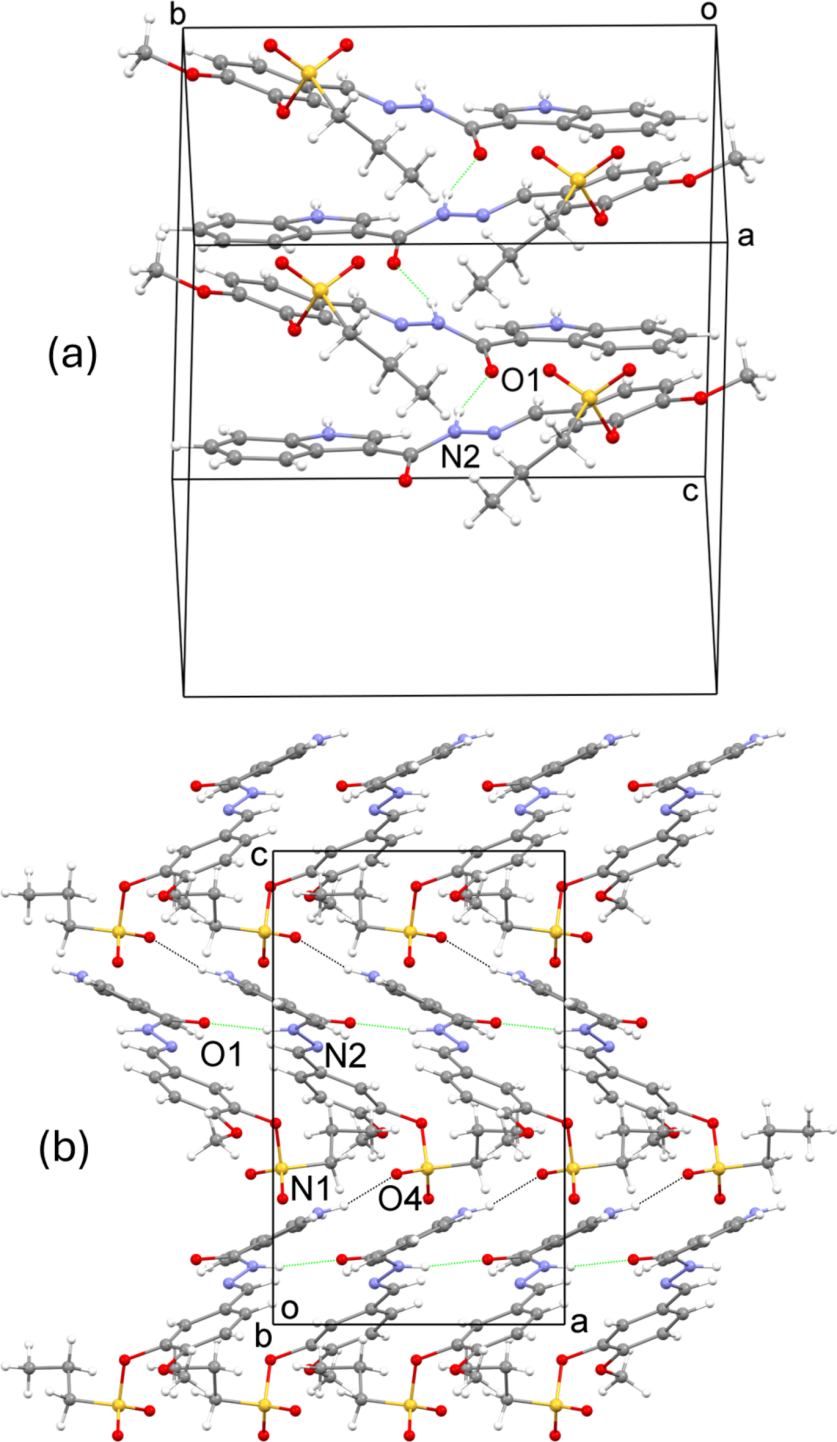
(*a*) A segment of the crystal structure showing mol­ecules linked through N—H⋯O hydrogen bonds (green dotted lines). (*b*) The crystal structure viewed down the *b* axis with C—H⋯O inter­actions shown as black dotted lines.

In the crystal, the mol­ecules are linked by N—H⋯O hydrogen-bonding inter­actions involving the N—H and carbonyls of methyl­ideneformohydrazide groups of adjacent mol­ecules. Thus, N2—H2*A*⋯O1 inter­actions link mol­ecules related by glide symmetry to form chains parallel to the *a* axis (Fig. 3 ▸*a*, Table 1 ▸). The chains are linked through N1—H1⋯O4 inter­actions involving the N—H group of the indolyl fragment and an oxygen atom of the sulfonate group to form a three-dimensional network (Fig. 3 ▸*b*, Table 1 ▸). O1 is also an acceptor to longer inter­molecular C—H⋯O inter­actions, namely C10—H10⋯O1 and C18—H18*A*⋯O1 (Table 1 ▸).

## 4. Database survey

A search of the CSD (version 5.46, November 2024; Groom *et al.*, 2016 ▸) for uncoordinated fragments of linked indolyl and methyl­ideneformohydrazide groups revealed *N′*-[(2-hy­droxy­phen­yl)methyl­idene]-1*H*-indole-3-carbohydrazide monohydrate (YODCIH; Chen & Yu, 2006 ▸), which has a similar twist angle to the title compound. The closest hit containing the sulfonate, meth­oxybenzene and methyl­ideneformohydrazide groups was (*E*)-2-meth­oxy-*N′*-(4-meth­oxy-3-(4-methyl­benzene­sulfon­yloxy)benzyl­idene)benzohydrazide ethanol solvate hemihydrate (HESRIH; Li *et al.*, 2024 ▸) in which the two planar fragments also have a twist angle (8.5°)comparable to the title compound.

## 5. Synthesis and crystallization

For synthesis of (*E*)-5-{[2-(1*H*-indole-3-carbon­yl)hydrazono]meth­yl}-2-meth­oxy­phenyl propane-1-sulfonate (**3**), a mixture of 10 mmol of 1*H*-indole-3-carbohydrazide (**1**) and 10 mmol of 5-formyl-2-meth­oxy­phenyl propane-1-sulfonate (**2**) in 20 ml of acetic acid/ethanol (1:2) was refluxed for 1 h. The mixture was filtered, and then the solid obtained was dried and recrystallized from ethanol. Yield: 91%; m.p. 485–486 K; Color: buff crystals; ^1^H-NMR (500 MHz, DMSO-*d*_6_) δ (ppm): 1.01 (*t*, 3H, *J* = 7.4 Hz, CH_2_CH_2_CH_3_), 1.86 (*m*, 2H, CH_2_CH_2_CH_3_), 3.46 (*t*, 2H, *J* = 7.6 Hz, CH_2_CH_2_CH_3_), 3.86 (*s*, 3H, OCH_3_), 7.13–7.19 (*m*, 2H, Ar-H), 7.22 (*d*, 1H, *J* = 8.6 Hz, Ar-H), 7.46 (*d*, 1H, *J* = 7.8 Hz, Ar-H), 7.59–7.64 (*m*, 2H, Ar-H), 8.21 (*br. s*, 3H, CH=N + Ar-H), 11.42 (*s*, 1H, NH), 11.76 (*s*, 1H, NH); ^13^C-NMR (126 MHz, DMSO-*d*_6_) δ (ppm): 12.38, 17.01, 51.45, 111.98, 120.80, 122.27, 122.65, 128.32, 133.86, 149.45; Analysis % for C_20_H_21_N_3_O_5_S (415.46). Calculated: C, 57.82; H, 5.10; N, 10.11. Found: C, 57.78; H, 5.18; N, 9.96.

## 6. Refinement

Crystal data, data collection and structure refinement details are summarized in Table 2 ▸. Hydrogen atoms were located in difference-Fourier maps. C-bound atoms were thereafter refined with restrained geometry using a riding model with displacement parameters constrained to either 1.2 or 1.5 times the equivalent isotropic displacement parameter of the parent C atom.

## Supplementary Material

Crystal structure: contains datablock(s) I. DOI: 10.1107/S2056989025002087/jp2017sup1.cif

Structure factors: contains datablock(s) I. DOI: 10.1107/S2056989025002087/jp2017Isup2.hkl

Supporting information file. DOI: 10.1107/S2056989025002087/jp2017Isup3.cml

CCDC reference: 2418615

Additional supporting information:  crystallographic information; 3D view; checkCIF report

## Figures and Tables

**Table 1:** Hydrogen-bond geometry (Å, °)

*D*—H⋯*A*	*D*—H	H⋯*A*	*D*⋯*A*	*D*—H⋯*A*
C10—H10⋯O1^i^	0.93	2.51	3.325 (4)	146
C18—H18*A*⋯O1^ii^	0.97	2.52	3.467 (5)	164
N2—H2*A*⋯O1^i^	0.86 (1)	2.22 (2)	3.048 (4)	163 (4)
N1—H1⋯O4^iii^	0.86 (1)	2.07 (3)	2.870 (5)	156 (5)

Symmetry codes: (i) 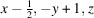; (ii) 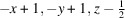; (iii) 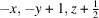.

**Table 2:** Experimental details

Crystal data	
Chemical formula	C_20_H_21_N_3_O_5_S
*M*_r_	415.46
Crystal system, space group	Orthorhombic, *Pca*2_1_
Temperature (K)	296
*a*, *b*, *c* (Å)	9.2969 (5), 14.0662 (7), 15.1168 (7)
*V* (Å^3^)	1976.85 (17)
*Z*	4
Radiation type	Mo *K*α
μ (mm^−1^)	0.20
Crystal size (mm)	0.34 × 0.14 × 0.09

Data collection	
Diffractometer	SuperNova, Dual, Cu at home/near, Atlas
Absorption correction	Gaussian (*CrysAlis PRO*; Rigaku OD, 2022 ▸)
*T*_min_, *T*_max_	0.618, 1.000
No. of measured, independent and observed [*I* > 2σ(*I*)] reflections	16123, 4696, 3791
*R*_int_	0.027
(sin θ/λ)_max_ (Å^−1^)	0.698

Refinement	
*R*[*F*^2^ > 2σ(*F*^2^)], *wR*(*F*^2^), *S*	0.043, 0.111, 1.05
No. of reflections	4696
No. of parameters	272
No. of restraints	3
H-atom treatment	H atoms treated by a mixture of independent and constrained refinement
Δρ_max_, Δρ_min_ (e Å^−3^)	0.23, −0.23
Absolute structure	Flack *x* determined using 1389 quotients [(*I*^+^)−(*I*^−^)]/[(*I*^+^)+(*I*^−^)] (Parsons *et al.*, 2013 ▸)
Absolute structure parameter	−0.05 (3)

Computer programs: *CrysAlis PRO* (Rigaku OD, 2022 ▸), *SHELXT* (Sheldrick, 2015*a* ▸), *SHELXL* (Sheldrick, 2015*b* ▸), *Mercury* (Macrae *et al.*, 2020 ▸) and *ORTEP-3 for Windows* (Farrugia, 2012 ▸).

## supplementary crystallographic information

### 5-{(E)-[(1H-Indol-3-ylformamido)imino]methyl}-2-methoxyphenyl propane-1-sulfonate Crystal data

**Table:**

C_20_H_21_N_3_O_5_S	*D*_x_ = 1.396 Mg m^−^^3^
*M**_r_* = 415.46	Mo *K*α radiation, λ = 0.71073 Å
Orthorhombic, *P**c**a*2_1_	Cell parameters from 7123 reflections
*a* = 9.2969 (5) Å	θ = 3.9–28.6°
*b* = 14.0662 (7) Å	µ = 0.20 mm^−^^1^
*c* = 15.1168 (7) Å	*T* = 296 K
*V* = 1976.85 (17) Å^3^	Block, colourless
*Z* = 4	0.34 × 0.14 × 0.09 mm
*F*(000) = 872	

### 5-{(E)-[(1H-Indol-3-ylformamido)imino]methyl}-2-methoxyphenyl propane-1-sulfonate Data collection

**Table:**

SuperNova, Dual, Cu at home/near, Atlas diffractometer	3791 reflections with *I* > 2σ(*I*)
Detector resolution: 10.5082 pixels mm^-1^	*R*_int_ = 0.027
ω scans	θ_max_ = 29.7°, θ_min_ = 3.6°
Absorption correction: gaussian (CrysAlisPro; Rigaku OD, 2022)	*h* = −12→11
*T*_min_ = 0.618, *T*_max_ = 1.000	*k* = −18→19
16123 measured reflections	*l* = −21→19
4696 independent reflections	

### 5-{(E)-[(1H-Indol-3-ylformamido)imino]methyl}-2-methoxyphenyl propane-1-sulfonate Refinement

**Table:**

Refinement on *F*^2^	Hydrogen site location: mixed
Least-squares matrix: full	H atoms treated by a mixture of independent and constrained refinement
*R*[*F*^2^ > 2σ(*F*^2^)] = 0.043	*w* = 1/[σ^2^(*F*_o_^2^) + (0.0461*P*)^2^ + 0.6044*P*] where *P* = (*F*_o_^2^ + 2*F*_c_^2^)/3
*wR*(*F*^2^) = 0.111	(Δ/σ)_max_ < 0.001
*S* = 1.05	Δρ_max_ = 0.23 e Å^−^^3^
4696 reflections	Δρ_min_ = −0.23 e Å^−^^3^
272 parameters	Absolute structure: Flack *x* determined using 1389 quotients [(*I*^+^)-(*I*^-^)]/[(*I*^+^)+(*I*^-^)] (Parsons *et al.*, 2013)
3 restraints	Absolute structure parameter: −0.05 (3)

### 5-{(E)-[(1H-Indol-3-ylformamido)imino]methyl}-2-methoxyphenyl propane-1-sulfonate Special details

**Table:**

Geometry. All esds (except the esd in the dihedral angle between two l.s. planes) are estimated using the full covariance matrix. The cell esds are taken into account individually in the estimation of esds in distances, angles and torsion angles; correlations between esds in cell parameters are only used when they are defined by crystal symmetry. An approximate (isotropic) treatment of cell esds is used for estimating esds involving l.s. planes.

### 5-{(E)-[(1H-Indol-3-ylformamido)imino]methyl}-2-methoxyphenyl propane-1-sulfonate Fractional atomic coordinates and isotropic or equivalent isotropic displacement parameters (Å^2^)

**Table:**

	*x*	*y*	*z*	*U*_iso_*/*U*_eq_	
C1	−0.0659 (4)	0.2094 (3)	0.7066 (3)	0.0495 (9)	
C2	−0.0849 (6)	0.1109 (3)	0.7088 (3)	0.0697 (13)	
H2	−0.168430	0.084058	0.731600	0.084*	
C3	0.0230 (6)	0.0556 (3)	0.6765 (3)	0.0715 (13)	
H3	0.013852	−0.010235	0.678105	0.086*	
C4	0.1460 (5)	0.0956 (3)	0.6413 (3)	0.0624 (11)	
H4	0.218451	0.055961	0.620438	0.075*	
C5	0.1645 (4)	0.1927 (2)	0.6362 (3)	0.0488 (8)	
H5	0.246976	0.218411	0.610950	0.059*	
C6	0.0574 (4)	0.2512 (2)	0.6696 (2)	0.0361 (7)	
C7	0.0362 (3)	0.3530 (2)	0.6756 (2)	0.0377 (7)	
C8	−0.0946 (4)	0.3660 (3)	0.7149 (3)	0.0496 (8)	
H8	−0.136701	0.424788	0.726252	0.059*	
C9	0.1332 (3)	0.4263 (2)	0.6439 (2)	0.0361 (6)	
C10	0.0879 (4)	0.6607 (2)	0.5743 (3)	0.0450 (8)	
H10	−0.007127	0.668033	0.592203	0.054*	
C11	0.1630 (4)	0.7397 (2)	0.5322 (2)	0.0415 (7)	
C12	0.3017 (3)	0.7294 (2)	0.4981 (2)	0.0378 (7)	
H12	0.349555	0.671639	0.503558	0.045*	
C13	0.3671 (3)	0.8037 (2)	0.4567 (2)	0.0377 (7)	
C14	0.2995 (4)	0.8918 (2)	0.4465 (2)	0.0420 (7)	
C15	0.1624 (4)	0.9023 (2)	0.4804 (3)	0.0488 (9)	
H15	0.114956	0.960221	0.475005	0.059*	
C16	0.0953 (4)	0.8267 (2)	0.5223 (3)	0.0502 (9)	
H16	0.002673	0.834642	0.544236	0.060*	
C17	0.3050 (5)	1.0491 (2)	0.3895 (3)	0.0609 (11)	
H17A	0.219977	1.038851	0.354718	0.091*	
H17B	0.368862	1.091027	0.358372	0.091*	
H17C	0.278991	1.077006	0.445144	0.091*	
C18	0.6978 (4)	0.6949 (3)	0.3417 (3)	0.0489 (9)	
H18A	0.728048	0.668857	0.285233	0.059*	
H18B	0.766370	0.743790	0.358143	0.059*	
C19	0.7020 (5)	0.6173 (4)	0.4099 (3)	0.0769 (14)	
H19A	0.691877	0.645464	0.468136	0.092*	
H19B	0.620706	0.575250	0.400642	0.092*	
C20	0.8367 (6)	0.5601 (4)	0.4077 (4)	0.0939 (18)	
H20A	0.841089	0.524917	0.353344	0.141*	
H20B	0.837658	0.516757	0.456748	0.141*	
H20C	0.918286	0.601747	0.411564	0.141*	
N1	−0.1539 (3)	0.2809 (3)	0.7350 (3)	0.0614 (9)	
N2	0.0700 (3)	0.5101 (2)	0.6228 (2)	0.0435 (6)	
N3	0.1519 (3)	0.58184 (19)	0.58674 (19)	0.0414 (6)	
O1	0.2641 (2)	0.41354 (16)	0.63705 (17)	0.0462 (6)	
O2	0.3750 (3)	0.96064 (16)	0.40450 (19)	0.0560 (7)	
O3	0.5089 (2)	0.79283 (17)	0.42481 (16)	0.0423 (5)	
O4	0.4236 (3)	0.6744 (2)	0.3176 (2)	0.0725 (9)	
O5	0.5327 (4)	0.8204 (3)	0.2653 (2)	0.0937 (12)	
S1	0.52915 (9)	0.74697 (7)	0.32886 (7)	0.0473 (2)	
H2A	−0.0209 (16)	0.520 (3)	0.622 (3)	0.051 (11)*	
H1	−0.243 (2)	0.280 (4)	0.750 (4)	0.097 (17)*	

### 5-{(E)-[(1H-Indol-3-ylformamido)imino]methyl}-2-methoxyphenyl propane-1-sulfonate Atomic displacement parameters (Å^2^)

**Table:**

	*U*^11^	*U*^22^	*U*^33^	*U*^12^	*U*^13^	*U*^23^
C1	0.0429 (19)	0.053 (2)	0.053 (2)	−0.0118 (17)	−0.0035 (16)	0.0162 (18)
C2	0.075 (3)	0.064 (3)	0.071 (3)	−0.028 (2)	−0.010 (2)	0.024 (2)
C3	0.100 (4)	0.040 (2)	0.074 (3)	−0.011 (2)	−0.020 (3)	0.010 (2)
C4	0.083 (3)	0.046 (2)	0.059 (2)	0.008 (2)	−0.006 (2)	−0.0061 (19)
C5	0.053 (2)	0.0437 (19)	0.050 (2)	0.0015 (16)	−0.0070 (17)	−0.0011 (16)
C6	0.0348 (16)	0.0397 (16)	0.0338 (15)	−0.0036 (13)	−0.0045 (12)	0.0050 (13)
C7	0.0310 (16)	0.0406 (16)	0.0413 (16)	0.0016 (13)	0.0001 (13)	0.0060 (14)
C8	0.0355 (18)	0.053 (2)	0.060 (2)	0.0052 (15)	0.0062 (15)	0.0079 (19)
C9	0.0322 (16)	0.0389 (16)	0.0372 (15)	0.0004 (13)	0.0009 (12)	−0.0007 (13)
C10	0.0342 (17)	0.0419 (17)	0.059 (2)	−0.0021 (14)	0.0086 (15)	0.0023 (16)
C11	0.0370 (18)	0.0369 (16)	0.0507 (19)	0.0002 (13)	0.0036 (14)	0.0003 (14)
C12	0.0362 (16)	0.0354 (15)	0.0418 (16)	0.0050 (12)	−0.0009 (14)	0.0015 (14)
C13	0.0324 (16)	0.0380 (16)	0.0425 (17)	0.0008 (12)	0.0019 (13)	−0.0037 (13)
C14	0.0404 (18)	0.0317 (15)	0.054 (2)	−0.0017 (13)	0.0034 (15)	−0.0004 (15)
C15	0.043 (2)	0.0325 (16)	0.071 (2)	0.0056 (14)	0.0048 (17)	0.0010 (16)
C16	0.0350 (18)	0.0430 (18)	0.072 (3)	0.0066 (15)	0.0111 (17)	−0.0013 (17)
C17	0.070 (3)	0.0339 (18)	0.079 (3)	0.0034 (17)	0.013 (2)	0.009 (2)
C18	0.0393 (18)	0.053 (2)	0.054 (2)	0.0074 (15)	0.0061 (15)	−0.0021 (17)
C19	0.079 (3)	0.089 (3)	0.063 (3)	0.033 (3)	0.008 (2)	0.021 (2)
C20	0.107 (4)	0.097 (4)	0.078 (3)	0.053 (3)	0.010 (3)	0.018 (3)
N1	0.0344 (18)	0.071 (2)	0.079 (2)	−0.0050 (16)	0.0147 (16)	0.0224 (19)
N2	0.0314 (14)	0.0394 (14)	0.0596 (17)	0.0005 (12)	0.0067 (13)	0.0095 (14)
N3	0.0343 (14)	0.0379 (14)	0.0521 (16)	−0.0026 (12)	0.0046 (12)	0.0034 (12)
O1	0.0297 (12)	0.0447 (12)	0.0642 (15)	−0.0001 (10)	0.0019 (11)	0.0073 (12)
O2	0.0526 (15)	0.0357 (12)	0.0796 (19)	0.0012 (10)	0.0126 (13)	0.0106 (13)
O3	0.0312 (11)	0.0469 (13)	0.0488 (13)	0.0007 (10)	0.0042 (9)	−0.0045 (11)
O4	0.0489 (16)	0.093 (2)	0.076 (2)	−0.0002 (15)	−0.0067 (15)	−0.0299 (18)
O5	0.126 (3)	0.092 (2)	0.064 (2)	0.052 (2)	0.020 (2)	0.0295 (19)
S1	0.0443 (4)	0.0555 (5)	0.0420 (4)	0.0126 (4)	0.0015 (4)	0.0018 (4)

### 5-{(E)-[(1H-Indol-3-ylformamido)imino]methyl}-2-methoxyphenyl propane-1-sulfonate Geometric parameters (Å, º)

**Table:**

C1—N1	1.365 (5)	C13—O3	1.412 (4)
C1—C2	1.398 (6)	C14—O2	1.354 (4)
C1—C6	1.405 (5)	C14—C15	1.382 (5)
C2—C3	1.360 (7)	C15—C16	1.386 (5)
C2—H2	0.9300	C15—H15	0.9300
C3—C4	1.381 (7)	C16—H16	0.9300
C3—H3	0.9300	C17—O2	1.422 (4)
C4—C5	1.378 (5)	C17—H17A	0.9600
C4—H4	0.9300	C17—H17B	0.9600
C5—C6	1.387 (5)	C17—H17C	0.9600
C5—H5	0.9300	C18—C19	1.503 (6)
C6—C7	1.447 (5)	C18—S1	1.742 (3)
C7—C8	1.367 (5)	C18—H18A	0.9700
C7—C9	1.451 (4)	C18—H18B	0.9700
C8—N1	1.354 (5)	C19—C20	1.488 (6)
C8—H8	0.9300	C19—H19A	0.9700
C9—O1	1.235 (4)	C19—H19B	0.9700
C9—N2	1.355 (4)	C20—H20A	0.9600
C10—N3	1.273 (4)	C20—H20B	0.9600
C10—C11	1.458 (5)	C20—H20C	0.9600
C10—H10	0.9300	N1—H1	0.858 (14)
C11—C16	1.385 (5)	N2—N3	1.376 (4)
C11—C12	1.397 (5)	N2—H2A	0.856 (13)
C12—C13	1.361 (5)	O3—S1	1.599 (3)
C12—H12	0.9300	O4—S1	1.426 (3)
C13—C14	1.398 (4)	O5—S1	1.411 (3)
			
N1—C1—C2	130.3 (4)	C16—C15—H15	119.9
N1—C1—C6	107.9 (3)	C11—C16—C15	121.6 (3)
C2—C1—C6	121.8 (4)	C11—C16—H16	119.2
C3—C2—C1	117.8 (4)	C15—C16—H16	119.2
C3—C2—H2	121.1	O2—C17—H17A	109.5
C1—C2—H2	121.1	O2—C17—H17B	109.5
C2—C3—C4	121.0 (4)	H17A—C17—H17B	109.5
C2—C3—H3	119.5	O2—C17—H17C	109.5
C4—C3—H3	119.5	H17A—C17—H17C	109.5
C5—C4—C3	121.9 (4)	H17B—C17—H17C	109.5
C5—C4—H4	119.0	C19—C18—S1	113.9 (3)
C3—C4—H4	119.0	C19—C18—H18A	108.8
C4—C5—C6	118.6 (4)	S1—C18—H18A	108.8
C4—C5—H5	120.7	C19—C18—H18B	108.8
C6—C5—H5	120.7	S1—C18—H18B	108.8
C5—C6—C1	118.8 (3)	H18A—C18—H18B	107.7
C5—C6—C7	135.0 (3)	C20—C19—C18	113.5 (4)
C1—C6—C7	106.1 (3)	C20—C19—H19A	108.9
C8—C7—C6	106.3 (3)	C18—C19—H19A	108.9
C8—C7—C9	126.9 (3)	C20—C19—H19B	108.9
C6—C7—C9	126.7 (3)	C18—C19—H19B	108.9
N1—C8—C7	109.9 (3)	H19A—C19—H19B	107.7
N1—C8—H8	125.0	C19—C20—H20A	109.5
C7—C8—H8	125.0	C19—C20—H20B	109.5
O1—C9—N2	122.3 (3)	H20A—C20—H20B	109.5
O1—C9—C7	122.4 (3)	C19—C20—H20C	109.5
N2—C9—C7	115.3 (3)	H20A—C20—H20C	109.5
N3—C10—C11	120.3 (3)	H20B—C20—H20C	109.5
N3—C10—H10	119.8	C8—N1—C1	109.7 (3)
C11—C10—H10	119.8	C8—N1—H1	117 (4)
C16—C11—C12	118.1 (3)	C1—N1—H1	131 (4)
C16—C11—C10	120.3 (3)	C9—N2—N3	119.5 (3)
C12—C11—C10	121.6 (3)	C9—N2—H2A	124 (3)
C13—C12—C11	120.2 (3)	N3—N2—H2A	115 (3)
C13—C12—H12	119.9	C10—N3—N2	116.1 (3)
C11—C12—H12	119.9	C14—O2—C17	117.6 (3)
C12—C13—C14	122.1 (3)	C13—O3—S1	117.6 (2)
C12—C13—O3	119.4 (3)	O5—S1—O4	117.3 (3)
C14—C13—O3	118.6 (3)	O5—S1—O3	108.98 (19)
O2—C14—C15	125.1 (3)	O4—S1—O3	108.43 (17)
O2—C14—C13	116.9 (3)	O5—S1—C18	111.2 (2)
C15—C14—C13	117.9 (3)	O4—S1—C18	109.37 (19)
C14—C15—C16	120.2 (3)	O3—S1—C18	100.09 (16)
C14—C15—H15	119.9		
			
C20—C19—C18—S1	168.2 (4)		

### 5-{(E)-[(1H-Indol-3-ylformamido)imino]methyl}-2-methoxyphenyl propane-1-sulfonate Hydrogen-bond geometry (Å, º)

**Table:**

*D*—H···*A*	*D*—H	H···*A*	*D*···*A*	*D*—H···*A*
C10—H10···O1^i^	0.93	2.51	3.325 (4)	146
C18—H18*A*···O1^ii^	0.97	2.52	3.467 (5)	164
N2—H2*A*···O1^i^	0.86 (1)	2.22 (2)	3.048 (4)	163 (4)
N1—H1···O4^iii^	0.86 (1)	2.07 (3)	2.870 (5)	156 (5)

Symmetry codes: (i) *x*−1/2, −*y*+1, *z*; (ii) −*x*+1, −*y*+1, *z*−1/2; (iii) −*x*, −*y*+1, *z*+1/2.
